# Overcoming Multidrug Resistance in Bacteria Through Antibiotics Delivery in Surface-Engineered Nano-Cargos: Recent Developments for Future Nano-Antibiotics

**DOI:** 10.3389/fbioe.2021.696514

**Published:** 2021-07-08

**Authors:** Xinfu Yang, Wenxin Ye, Yajun Qi, Yin Ying, Zhongni Xia

**Affiliations:** ^1^Department of Pharmacy, Tongde Hospital of Zhejiang Province, Hangzhou, China; ^2^Department of Urology, Tongde Hospital of Zhejiang Province, Hangzhou, China; ^3^Department of Pharmacy, The Cancer Hospital of the University of Chinese Academy of Sciences (Zhejiang Cancer Hospital), Hangzhou, China; ^4^Institute of Cancer and Basic Medicine (IBMC), Chinese Academy of Sciences, Hangzhou, China; ^5^College of Pharmaceutical Sciences, Zhejiang University, Hangzhou, China

**Keywords:** antibiotics, multi-drug resistance, nanocarriers, surface engineering, targeted delivery, enhanced efficacy

## Abstract

In the recent few decades, the increase in multidrug-resistant (MDR) bacteria has reached an alarming rate and caused serious health problems. The incidence of infections due to MDR bacteria has been accompanied by morbidity and mortality; therefore, tackling bacterial resistance has become an urgent and unmet challenge to be properly addressed. The field of nanomedicine has the potential to design and develop efficient antimicrobials for MDR bacteria using its innovative and alternative approaches. The uniquely constructed nano-sized antimicrobials have a predominance over traditional antibiotics because their small size helps them in better interaction with bacterial cells. Moreover, surface engineering of nanocarriers offers significant advantages of targeting and modulating various resistance mechanisms, thus owe superior qualities for overcoming bacterial resistance. This review covers different mechanisms of antibiotic resistance, application of nanocarrier systems in drug delivery, functionalization of nanocarriers, application of functionalized nanocarriers for overcoming bacterial resistance, possible limitations of nanocarrier-based approach for antibacterial delivery, and future of surface-functionalized antimicrobial delivery systems.

## Introduction

Multidrug resistance (MDR) is a type of insensitivity developed by microorganisms to lethal doses of antibiotics. MDR has become a major concern for antibiotics regarding their efficacy against pathogenic diseases (Desselberger, 2000). The statistics of infections caused by MDR bacteria show that the insensitiveness of bacteria toward antibiotics has risen many folds in recent years. Several reports have emphasized that the resistance faced by antimicrobials is a big risk to human health in Europe (Llor and Bjerrum, 2014). It is claimed that Europe would spend €1.5 billion annually of its economy to meet the rate of mortality caused by the MDR bacterial infections (Rémy et al., 2015). On the other hand, in the US, more than two million people suffer from the MDR bacterial infections yearly and become the death cause of about 23,000. Similarly, these diseases are responsible for a collective $55 billion additional annual societal and healthcare expenses in the US (Prestinaci et al., 2015). The unavailability of the development of new antibacterial agents has further worsened the position of MDR bacteria (Li and Webster, 2018). Moreover, the treatment of MDR bacteria with ineffective antibiotics further supports the expansion of tolerance in bacteria. For example, 40–60% of *Staphylococcus aureus* strains collected from different US hospitals are resistant to methicillin and in certain cases even resistant to vancomycin and carbapenems (Ventola, 2015).

Conventional drug delivery systems (DDSs) do not possess the potential to address this issue and display limitations. The ineffectiveness of antibiotics is not the only reason, but low bioavailability, inadequate access to spots of infection, and the growth of MDR bacteria are also equally responsible (Canaparo et al., 2019). Thus, there is an increasing need for designing and developing novel delivery strategies for enhancing therapeutic efficacy of currently available antibiotics, especially against MDR bacteria. Nanotechnology is a developing field of science dealing with materials at nano- or molecular levels. It is presently making exceptional wonders in basic and applied sciences, such as biology, medicines, chemistry, and physics (Roco, 2003). Nanomedicine has diverse applications in biological and biomedical sciences for both diagnosis and drug delivery. Nanotechnology has provided a multipipeline platform to develop nanoparticle (NP)-based biomolecular sensors (Miranda et al., 2010), cancer therapies (Portney and Ozkan, 2006; Kennedy et al., 2011), targeted drug carriers (Hallaj-Nezhadia et al., 2010; Falanga et al., 2011), and rapid recognition of pathogens (Daaboul et al., 2010; Cao et al., 2011). The physical and chemical characteristics of materials are made completely different upon bringing them to nanosize. The increased surface area is actually responsible for the changes in these properties as it improves the reactivity of materials at the nanoscale (Gu et al., 2003; Ahmad et al., 2006).

The NPs offer an ultimate solution to address the MDR bacteria as they not only be used as carriers for natural antimicrobial and antibiotics but also fight against bacteria themselves (Wang et al., 2017). NP-based DDSs have the capability to deliver a broad range of therapeutics to the site of infection safely and effectively either contained within the structure or bound to their surface (Pissuwan et al., 2011; Gholipourmalekabadi et al., 2017). Moreover, the unique physiochemical properties of NPs make them potential candidates to be used for superior therapeutic efficacy against MDR bacteria (Naskar and Kim, 2019). NPs can execute their action *via* numerous bactericidal routes, getting it complicated for bacteria to develop resistance against them (Hajipour et al., 2012). These bactericidal routes are reliant upon the size, shape, fundamental core material, and surface chemistry of NPs. Moreover, high antibiotics loading combined with high penetrating ability into biological membranes make NPs distinguishable for the transportation of antibiotics. In addition, the ability to modify NPs interaction with bacteria cell wall or membrane plays a key role in enhancing the efficacy of the nanocarriers and treatment (Goodman et al., 2004; Gupta et al., 2016).

The development of resistance to antibiotics by bacteria not only decreases the drug's therapeutic efficacy for the treatment of life-threatening infectious diseases but also increases the overall cost of treatment strategies. Overcoming antibiotic resistance in bacteria has been one of the challenging tasks for biomedical scientists. Currently, surface-functionalized nanocarriers are widely used for overcoming MDR in bacteria against antibiotics. This review focuses on mechanisms of MDR development in bacteria and the current trends in nanocarrier-based antibiotics delivery for overcoming MDR in bacteria.

## Health-Related Problems and Financial Burden Associated with MDR

**Figure 1:**
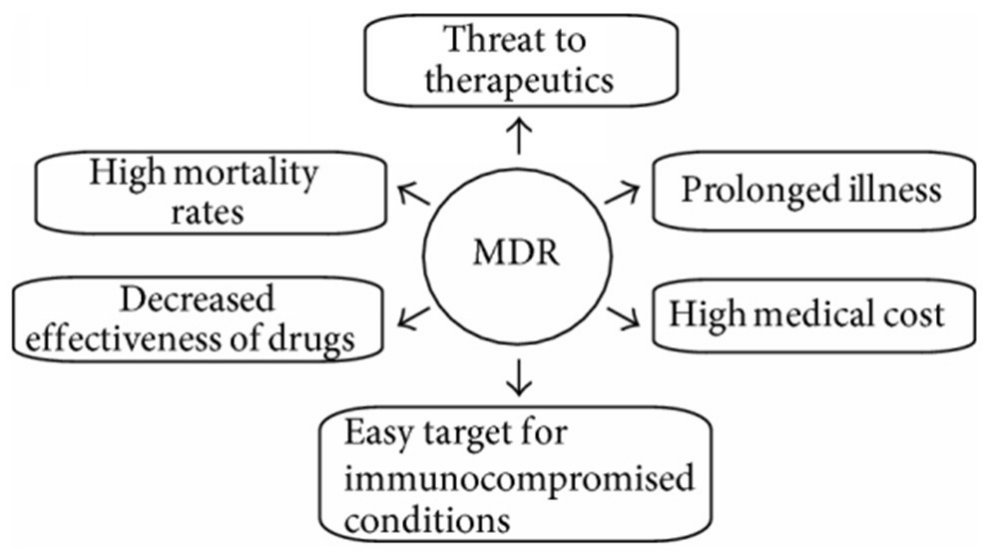
Various social- and health-related issues with multidrug-resistant bacteria (Tanwar et al., 2014).

The resistance of microbial is associated with high medical costs and high mortality rates and has a substantial influence on the efficacy of antimicrobial agents (Figure 1). MDR gives rise to a barrier in controlling the diseases by increasing the chance of proliferating resistant pathogens, therefore, worsening the effectiveness of treatment and, thus, following an extended time of infection in patients (Wang et al., 2020). The efficacy of antimicrobial agents is considerably affected by the quality of public hygiene and variation in the resistance profiles of fungal and bacterial pathogens. The treatment cost has also risen as the pathogens became resistant to existing medications, which led to more expensive therapies. The success of currently using medical applications, such as cancer chemotherapy and organ transplantation, has also contributed significantly to the development of MDR. Expansion of global tourism and trade results in the spread of high-potential MDR pathogens across the world and a decline in import–export of numerous products disturbing the financial system of developing countries (Fishbain and Peleg, 2010; Tanwar et al., 2014; World Health Organization, 2014).

## Multidrug Resistance Development in Bacteria: Mechanisms at Cellular and Molecular Levels

The knowledge of microbial genetics and the mechanism of genetic manipulation will give greater insight and offer a different aspect into combatting the resistance mechanisms (Hayes and Wolf, 1990). The resistance mechanisms can be categorized into intrinsic, which is linked to the integral and inherent property of the microbes and acquired one (Cox and Wright, 2013).

### Intrinsic Resistance

**Figure 2:**
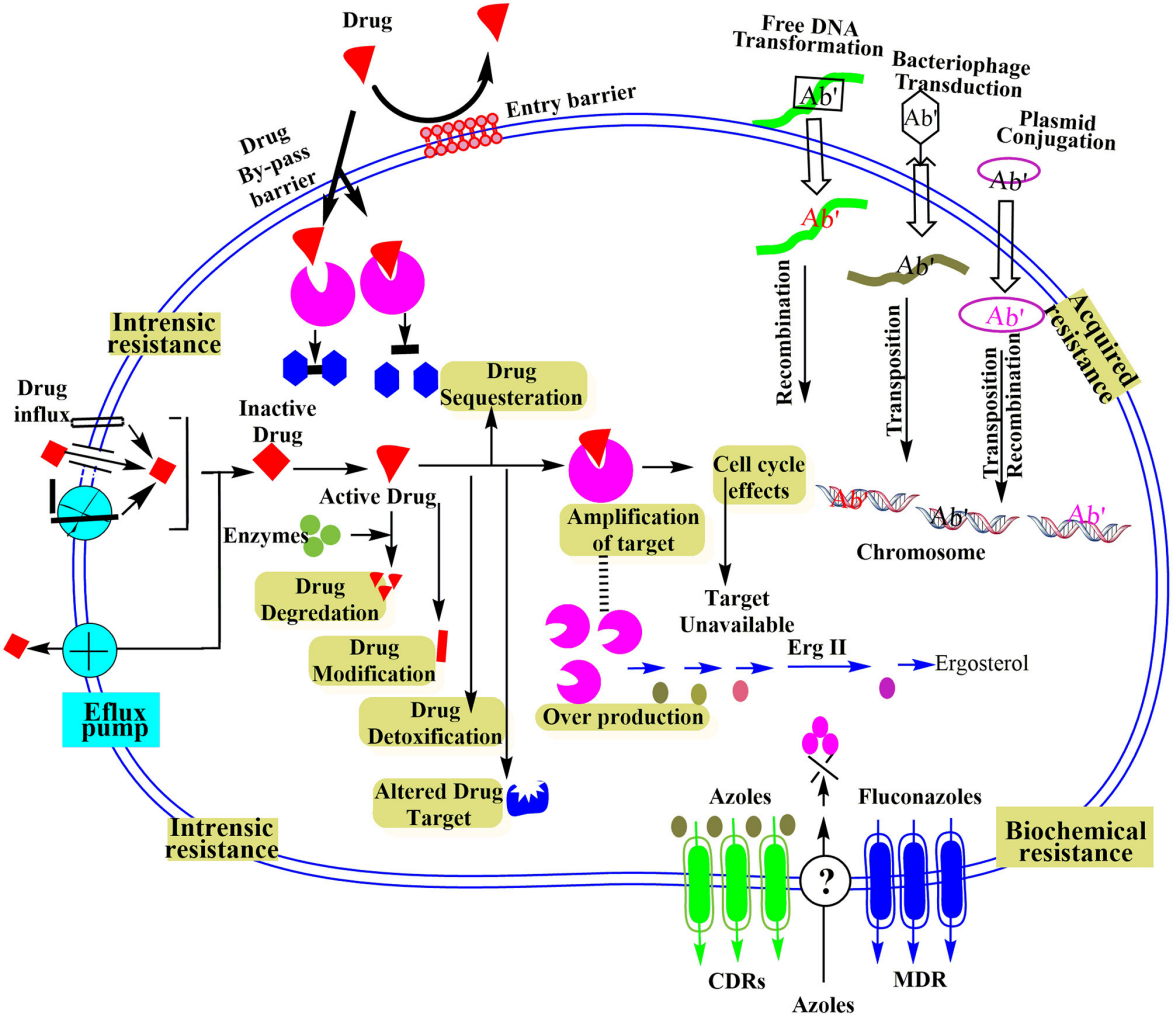
Schematic illustration of different microbial resistance mechanisms at molecular level reproduced from Ray et al. (2017).

Intrinsic resistance at a molecular level is based on integral or inherent properties of bacteria that are evolutionarily developed to resist the antimicrobial agents. The natural resistance characteristic of microbes sometimes experiences natural genomic alternations owing to the lack of antibiotic-based selective stress (Cox and Wright, 2013). Though generally, the antimicrobial-based microecological stress causes the inducement for pathogen adaptation. Evolutionary competition or mutations make it possible to get drug resistance gene, and it could occur because of some particular events as drawn in Figure 2 and described below:

#### Modification/Absence of Target Site

The uptake of antimicrobial agents by microbes is an important step for a target-oriented action. Microbial membrane contains beta barrel proteins (porins), which assist antimicrobial agents to pass the bacterial cell membrane. Some bacteria could control their outer membrane to defend themselves from external antimicrobial agents. For instance, some Gram-negative bacteria can alter the membrane porin selectivity, frequency, and size to minimize the uptake of specific antibacterial agents like aminoglycosides. Similarly, the variation in the penicillin-binding protein (PBP) site results in the insensitivity to antibiotics containing the β-lactam moiety (Malouin and Bryan, 1986).

#### Species-Specific Structure of Target Site

It is somehow clear that the mode of action of antimicrobial agents is almost similar in the same bacterial communities. However, species specificity has been a factor for the low affinity of antibiotics to its target site in some cases. Even within a single genus, different species of bacteria can modify the binding site of the antibiotic by producing several structural shapes for the same target and acquire resistance. For example, in *S. aureus* the large ribosomal subunit has specific binding modes and structural shapes for different antibiotics (Russell, 2002).

#### Inactivation of Antimicrobial Agents

One of the efficient practices implemented by microbes for their protection is to manipulate or destroy the active component of the therapeutic agent. For example, in cephalosporins and penicillins, the active β-lactam ring is converted into inactive penicilloic acid *via* hydrolyzing with an enzyme β-lactamase. The antibiotics then inept to bind with the PBPs, and the cell membrane of bacteria is protected from damage (Waxman and Strominger, 1983). Several Gram-positive and Gram-negative bacteria have been found that have adopted such type of inactivation against aminoglycosides, chloramphenicol, etc. *via* adenylation, phosphorylation, and acetylation (Bockstael and Van Aerschot, 2009).

#### Presence of Efflux Pumps

An antimicrobial agent requires to be inside a microbial system for a longer period in high concentrations to exert a lasting effect. However, certain bacteria have highly effective drug efflux pumps that expel out the drug thus leaving an inadequate amount of the drug for proper action. Even some pumps selectively force out specific antibiotics, such as tetracyclines, streptogramins, lincosamides, and macrolides, whereas MDR pumps extrude a variety of functionally and structurally different drugs (Lewis, 1994). The sodium and proton motive forces motivate the proteins of all efflux families except for ATP-binding cassette (ABC) transporters to perform the efflux activities. In contrast, the ATP hydrolysis pushes the primary ABC transporters to accomplish the efflux activity. These tactics have been detected in: (a) *Streptococcus pneumoniae* and *S. aureus* against fluoroquinolones, (b) *Staphylococci* against streptogramins and macrolides, (c) Enterobacteriaceae against chloramphenicol (d) *Escherichia coli* and other Enterobacteriaceae against tetracyclines.

#### High Neutralizing Capacity

Some of the bacteria produce toxic compounds to keep themselves safe from their competitors and predators. At the same time, they also intend to escape from the destructive effects of these toxic chemicals they discharge (Frère, 1995). This can be observed in the case of *Streptomyces* spp. and others, which are used to produce antibiotics. These bacteria develop resistance including the inactivation of their own antibiotics, such as neomycin and streptomycin by acetyltransferases and phosphotransferases. *Saccharopolyspora erythraea* (formerly *Streptomyces erythraeus*), erythromycin-producing bacterium, protected the target site, that is, rRNA by methylation to keep itself safe.

#### Low Drug Concentration

The insufficient drug concentration inside the host system is due to less time for circulation, fast metabolism, low bioavailability, and instability of the drug. All these factors potentially contribute to low drug concentration at the focus site, but as the drug is subjected to the biological milieu so the microbe or the tumor cells can develop resistance (Gullberg et al., 2011).

#### Stress Response

Other environmental factors, such as oxidative stress, anoxia, viral infection, trauma, heat, UV irradiation, osmotic sock, and pH, are also involved in genetic mutations of the cell. These mutations provide resistance to the stress factors and to antimicrobial agents. There are generally four stress-induced regulons in prokaryotes, namely, the response to alkylating agents, the heat-shock response, the oxyR network, and the SOS response. For example, the dnak and groEL heat-shock proteins in the *E. coli* are induced by UV irradiation, hyperthermia, and even nalidixic acid. In *Salmonella* Typhimurium, cell develops the ability to adjust the H_2_O_2_-induced oxidative stress, which also provides resistance against heat killing (Poole, 2012).

### Acquired Resistance

The acquired resistance mechanism comprises gene exchange/transfer methods or genetic mutation *via* the process of conjugation, transduction, or transformation (Flintoff, 1989; Frost et al., 2005). After the transfer of the resistance gene, the mechanistic biological activity or gene overexpression modifies the drugs in such a way as to nullify its effect. For instance, the mutated LexA repressor gene in *E. coli* has an influence in the SOS signals regulation (Little and Mount, 1982). Moreover, *S*. Typhimurium modifies the expression of several stress-regulating genes, such as glutathione peroxidase, SOD, catalase, etc. when resistant to H_2_O_2_.

### Chromosomal-Based Genetic Alteration

A modification of drug targets is one of the most adaptive mechanisms by microbes to develop acquired resistance. The resistance mechanism against fluoroquinolone can be attributed to efflux pump machinery and genetic alterations (Hooper and Jacoby, 2015). The modification of drug targets, such as topoisomerase IV and DNA gyrase, confers fluoroquinolone resistance. The role of these targets is critical in DNA duplication as each target comprising of two subunits: GrlA/ParC and GrlB/ParE for topoisomerase IV and GyrA and GyrB for DNA gyrase. One unit of these targets carries up the hydrolysis and ATP-binding role, whereas the other function for the DNA-binding role. The mutational modification in the region of DNA-binding domain, which regulates quinolone-resistance confers antibiotic resistance. Multiple mutations introduce additive effects to increase the resistant features of bacterium. Similarly, rifamycins can be administered for tuberculosis infection as a front-line therapeutic either alone or in combination with streptomycin, isoniazid, etc., whereas the mutation of RpoB point deters the binding affinity of the drugs at the subunit of RNA polymerase conferring combinative drug resistance (Mariam et al., 2004). The modification of dihydropteroate synthase, sulfonamide targets, results in lower enzymatic activity for the drug. The mutation of dihydrofolate reductase by blocking with trimethoprim results in protein overinduction and lower drug affinity. Point mutations at 23S rRNA and 16S rRNA operons confer macrolide, lincosamide, and streptogramin (MLS) antibiotics and tetracycline antibiotic resistance (Ross et al., 1998).

#### Genomic Duplication

Gene mutations primarily comprise of gene mutational events accompanied by gene overexpression or amplification. The process of genomic duplication is relatively predominant in eukaryotic cells to confer drug resistance. This genetic induction results in variation at the protein level, which enhances the biosynthetic machinery and overexpresses many transporters. In *E. coli*, the exposure of tetracycline imposes the genomic amplification of acrAB locus, which facilitates the efflux pump systems of acrAB, leading to MDR phenotype (Nikaido and Zgurskaya, 2001). Similar duplication process has also been reported in *S. aureus* for methicillin resistance. The amplification of genome makes one of the resistance mechanisms that prevent the restrictions of mutational aspects. However, it has been well-established that the microbes return to their typical phenotypes in the absence of drugs. Therefore, it can be concluded that this tolerance mechanism is temporary.

#### Modulated Drug Targets

*Staphylococcus aureus*, β-lactamase-producing microbe, was the first methicillin- and penicillin-resistant strain. The resistance mechanism involved in producing changes in PBPs *via* genetic mutations that confer β-lactam resistance in *Staphylococcus* and *Streptococcus*. The mecA encodes this gene on a movable genetic unit in resistant *S. aureus* (Wielders et al., 2001). In resistant *S. aureus*, PBP2 enzyme behaves bifunctionally where the transglycosylase and transpeptidase activities switch in according to the exposure of drugs for conveying tolerance or susceptibility features of bacterium (Brown and Reynolds, 1980). The plasmid-borne Qnr elements, which significantly suffer fluoroquinolone sensitivity, are found usually in Gram-negative species of non-typhoidal *Salmonella, Shigella, E. coli*, etc. (Piekarska et al., 2012). The MfpA and Qnr (pentapeptide repeat proteins) modulate fluoroquinolone resistance by protecting topoisomerase II and DNA gyrase, respectively. Qnr also shields topoisomerase from drugs effect. Besides, MfpA in *Mycobacterium* interacts with DNA gyrase producing a similar structural outlook and activity as exhibiting by B-DNA-inhibiting ciprofloxacin (Montero et al., 2001). MfpA and Qnr can strengthen the resistance profile to advance level by coupling with other modes.

#### Efflux Mechanisms and Membrane Permeability Channel

**Figure 3:**
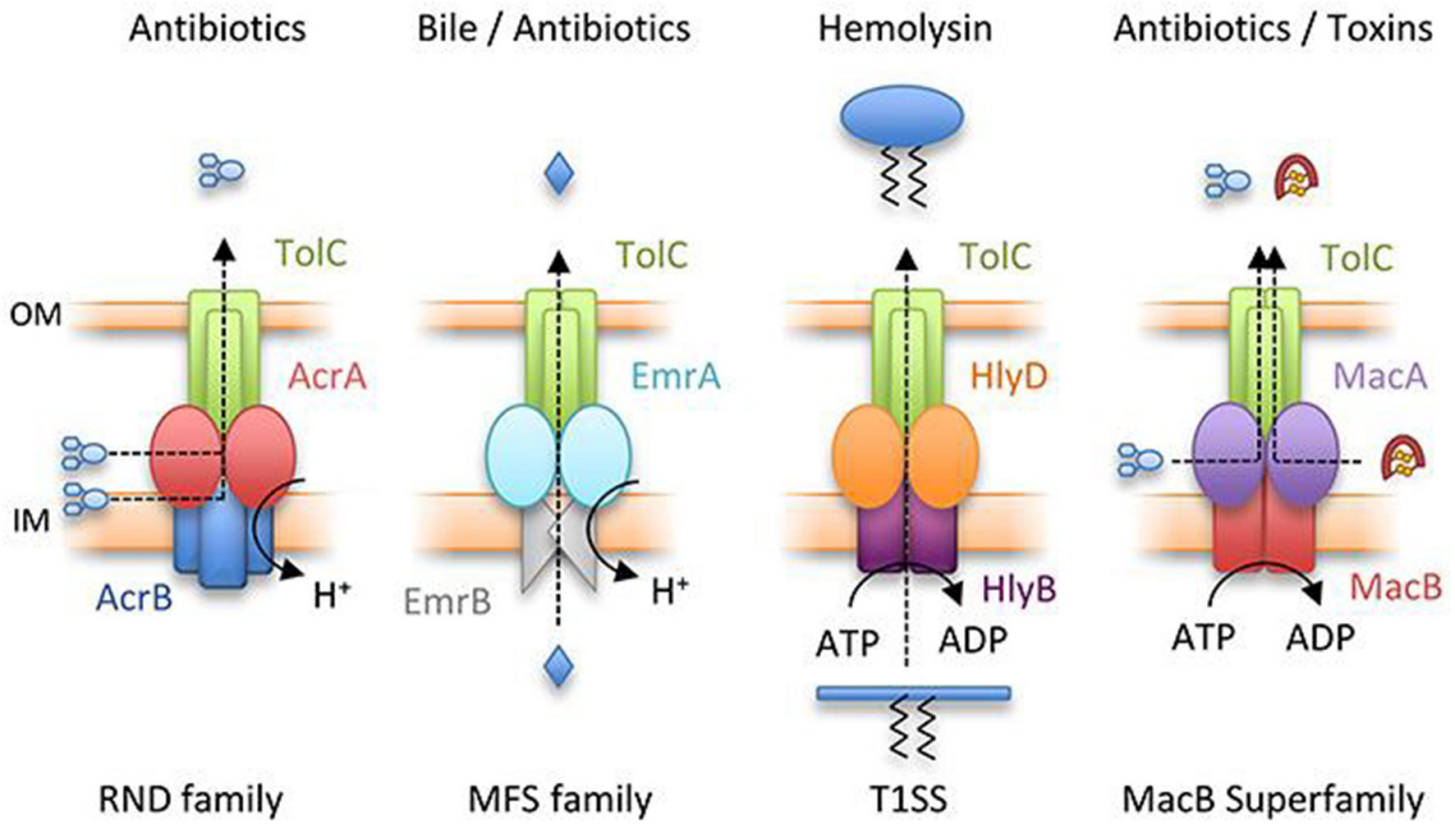
Representation of major classes of tripartite efflux pump. RND family pump, AcrAB-TolC; MFS pump, EmrAB-TolC; T1SS, HlyBD-TolC; MacB superfamily pump, MacAB-TolC. OM and IM represent outer and inner membranes, respectively (Greene et al., 2018) with permission. MFS, major facilitator superfamily; RND, resistance-nodulation-cell division; T1SS, type I secretion system.

Instead of restricting to drug internalization and uptake, resistance is generally due to the low or insignificant interaction of the drug with cellular targets because of drug effusion. The drug is kicked out from the cell by efflux pump mechanism (Figure 3) and was initially detected in the development of tetracycline resistance (Poole, 2005). The efflux mechanism is driven with ATP hydrolysis by the ABC group of primary transporters; however, the remaining secondary groups take part in conferring drug expulsion by proton-motive gradient force (Kobayashi et al., 2001). The proteins involve in the drug expulsion are either classified into single component systems bearing a specific substrate range or two protein systems to enable the binding of different structural compounds leading to a broad resistance phenotype spectrum. Resistance-nodulation-cell division (RND) transporters facilitate outflow of cytosolic proteins *via* the outer and inner membrane barriers. The efflux transporters of tetracycline consist of transmembrane spanning regions, which provide space to regulate the protein expression through the transcriptional repressor. The expression of efflux machinery of tetracycline is promoted by the deactivation of the repressor with the drugs.

## Surface Engineered Nano-Cargos: Novel Tools for Effective Drug Delivery

Nanocarriers are getting increased interest among researchers working on nanomedicines and pharmaceutical formulations owing to their unique features. Nanocarriers, that is, nanocapsules and polymeric NPs comprising of non-toxic biodegradable polymers, organic/inorganic nanomaterials, solid-lipid NPs, liposomes, etc. are among the most practiced carriers for different drugs (Patra et al., 2018). In fact, ocular, injectable, oral, and transdermal formulations mostly consist of the aforesaid nanomaterials exhibiting promising features, including higher intracellular penetration, high drug-loading capacity, specific targeting, long circulation in blood, and so on (Valavanidis and Vlachogianni, 2016). In the last few decades, extensive work has been done in developing advanced, less toxic, safer, and novel nanocarriers with tunable properties. Nanocarriers can be made multifunctional by incorporating certain chemical substances, such as peptides and vitamins through coupling chemistry or coating inorganic NPs with biocompatible materials. Such functionalization can improve some important features of nanocarriers, such as intracellular penetration, environmental friendliness, biocompatibility, and even targetability. Some intelligent and versatile carriers have been designed as innovative drug delivery nanocarriers, including polymer-based formulations, such as emulsions, hydrogels, virus-like materials, dendrimers, micelles, and polymeric NPs, lipid-based carriers, such as solid-lipid NPs, liposomes, and niosomes, carbon-based carriers, such as graphenes and nanotubes, and inorganic NPs, such as silica, silver, zinc oxide (Jain et al., 2020), and gold.

**Figure 4:**
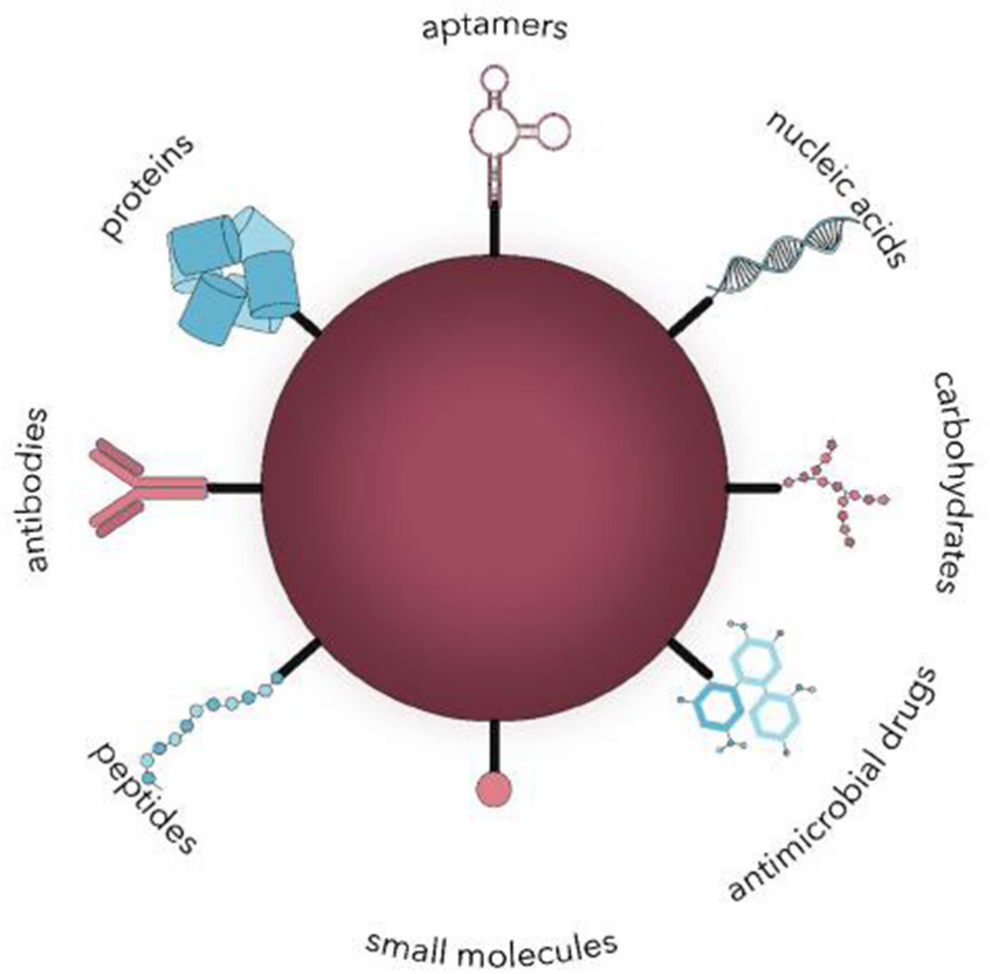
Surface functionalization of nanocarriers with different ligands for active microbial targeting (Spirescu et al., 2021).

Polymeric-based nanocarriers provide an interesting platform for the delivery of drugs owing to their exceptional physiochemical properties that stem from a size range that is analogous to and compatible with cellular and biomolecular systems. The interaction of nanocarriers with the biological environment can be determined from the surface nature of NPs (Auría-Soro et al., 2019). Therefore, the objective of surface functionalization of NPs is to make effective these interactions to decrease off-target drug exposure and maximize the drug circulation half-life. The strategies of surface functionalization can be categorized based on the nature of the ligand that is being conjugated as shown in Figure 4:

(i) Surface functionalization with small molecules, including vitamins, steroids, and monosaccharides or oligosaccharides.
(ii) Surface functionalization with macromolecules, including lipids, antibodies, polysaccharides, peptides, and aptamers (nucleic acids).
(iii) Surface functionalization with hydrophilic polymers.

Macromolecules, such as aptamers (nucleic acids), lipids, peptides, and polysaccharides, are mostly used as targeting ligands because of their selective affinity for certain sites. Generally, polymeric nanocarriers can be functionalized with biomolecules either directly *via* chemical bonds between the nanocarriers surface and biomolecules or by non-covalent physisorption. Polysaccharides have some advantages over other macromolecules such as well-established functionalization schemes, easy availability, and high biocompatibility (Doh and Yeo, 2012). Polysaccharides, such as heparin, hyaluronic acid (HA), chitosan (CS), and dextran, can offer steric protection against macrophage uptake and protein adsorption and are considered as stealth-coating matrixes (Abd Ellah Noura and Sara, 2017). An extensive literature exists, which describes the active targeting features of polysaccharides, such as chondroitin sulfate (Doh and Yeo, 2012), and HA (Xiao et al., 2015), and CS (Sheng et al., 2015).

Aptamers are short single-stranded oligonucleotides that can interact with cellular targets, such as transmembrane proteins, nucleic acids, or sugars, with high selectivity and affinity by getting well-defined three-dimensional secondary and tertiary structures (Catuogno et al., 2016). They have some distinct advantages over conventional antibodies like higher *in vivo* stability, lack of immunogenicity, higher ratio of target accumulation, smaller size, and easy of isolation (Lao et al., 2015). However, the absence of an Fc region restricts their half-life in circulation compared with that of an antibody, whereas this is not a major barrier when applying the aptamer as a targeting ligand.

Polymeric NPs coated with lipid offer several advantages in drug delivery, such as low cytotoxicity, extended half-life, better target specificity, and ease of surface engineering (Hadinoto et al., 2013). Natural phospholipids, including phosphatidylethanolamine, phosphatidic acid, phosphatidylserine, phosphatidylinositol, phosphatidylcholine, and phosphatidylglycerol and their synthetic counterparts {e.g., 1,2-dipalmitoyl-3-trimethylammonium-propane (DPTAP), 1,2-dilauroyl-sn-glycero-3-phosphocholine (DLPC), 1,2-dipalmitoyl-sn-glycero-3-phosphocholine (DPPC), N-[1-(2,3-dioleoyloxy)propyl]-N,N,N-trimethylammonium methyl-sulfate (DOTAP), 1,2-distearoylphosphatidylethanolamine (DSPE), and N (methylpolyoxyethylene oxycarbonyl)-1,2-distearoyl-sn-glycero-3-phosphoethanolamine (DSPE-PEG)}, have been mostly used for the surface coating of polymeric NPs (Mandal et al., 2013).

The use of antibodies as targeting ligands is promising because of their exceptional target affinity and specificity. The conjugation of antibodies to polymer NPs can impart high target recognition capability to nanocarriers. However, appropriate coupling of the antibody to the surface of NPs in proper orientation while preventing aggregate formation is essential for effective functionalization. A suitable chemistry must be introduced to antibody molecules and the surface of NPs for the covalent coupling of antibodies to the surface of nanocarriers. Functional group chemistry, like sulfhydryl (cysteine), carboxy (glutamic acid and aspartic acid), and amino (lysine), are the most frequently used functional groups in antibodies. The surface functionalization of NPs with antibodies *via* physical adsorption has also been investigated and found very effective in certain cases.

An important aspect of an effective drug delivery system is its ability to stay for a prolonged period in the circulation system. Phagocytic uptake by macrophages is one of the big challenges for extended circulation as it is the primary mechanism of particle clearance from the circulation system. When the surface of NPs functionalized with hydrophilic polymers such as polyethylene glycol (PEG) (2–20 kDa), it can extend the circulation of NPs (Knop et al., 2010). The hydrophilic polymers provide a steric barrier and delay opsonization or plasma proteins adsorption, which is a key step in phagocytic uptake of NPs (Knop et al., 2010). The non-specific interactions of NPs with blood components become least and decreased blood clearance of NPs. Moreover, PEG improves the stability of NPs in aqueous dispersions and prolongs its storage by reducing the aggregation tendency of particles (Suk et al., 2016). Although surface modification of nanocarriers *via* PEGylation has effectiveness in prolonging NP circulation after intravenous administration, but its coating on the nanocarriers can reduce the effective interaction with target cells. This is referred to as the “PEG dilemma.” The surface functionalization of NPs with receptor- or cell-specific ligand (in addition to PEG) can overcome the PEG dilemma. The PEG presence can also lead to the development of anti-PEG antibodies and hypersensitivity reactions in certain cases. In addition to PEG, other synthetic hydrophilic polymers, such as polyvinyl alcohol (PVA), poloxamers, polyaminoacids, and polyvinylpyrrolidone (PVP), have also been used as stealth coatings (Knop et al., 2010).

## Smart Nanosystems for Antibiotics Delivery

**Figure 5:**
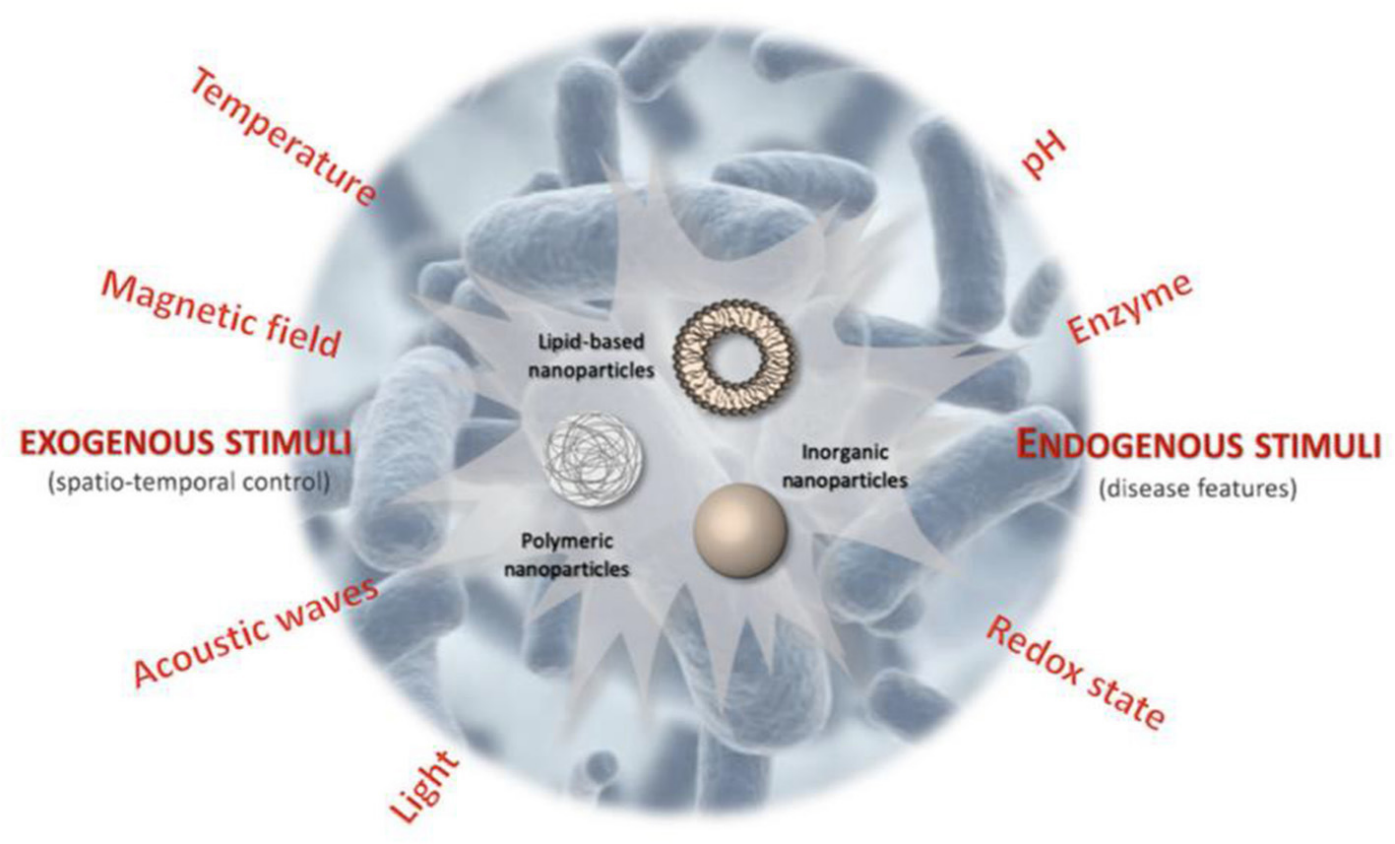
Schematic illustration of stimuli-responsive antibiotic drug delivery systems (Canaparo et al., 2019).

In the last few decades, nanotechnology has achieved unprecedented progress, and conventional DDSs have been shifted to smart DDSs with stimuli-responsive features. Taking the advantages of the response to specific external or internal triggers, these smart nanocarriers can improve the drug targeting efficacy and decrease toxicities or side effects of drugs (Liu et al., 2016). Specific exogenous or endogenous stimuli, that is, variations in electric or light pulses, ultrasound intensity, magnetic field, temperature or endogenic stimuli, that is, change in redox gradients, enzyme concentration, or pH triggers the release of the drugs from smart nanocarriers (Figure 5) (Mura et al., 2013). The pH change has been broadly exploited to switch the delivery of drugs to specific organs (i.e., the vagina or the gastrointestinal tract) and intracellular compartments (such as lysosomes or endosomes). These nanocarriers can release drugs when subtle environmental fluctuations are linked with pathological situations, such as infection or cancer. There are two main strategies to achieve this stimuli-responsive release *via*: (i) the development of polymeric systems with acid-labile bonds whose cleavage or alteration enables the release of payloads that are anchored to the polymer backbone; and (ii) the use of polymers (polybases or polyacids) with ionizable groups that undergo solubility and/or conformational changes in response to environmental pH variation and subsequently release the payload (Mura et al., 2013).

Temperature has also been extensively explored stimuli for DDSs in different fields, cancer being on top. Such DDSs are composed mainly of polymers that possess thermo-responsive blocks, which experience a sharp alteration in their aqueous solution properties (Lombardo et al., 2004; Gandhi et al., 2015) thus interrupt the nano-system structure and letting controlled release of the payload (Ward and Georgiou, 2011; Gandhi et al., 2015). Certain polymers are partially soluble below a specific temperature, known as lower critical solution temperature (LCST), where the polymer holds water molecules by making hydrogen bonds. Above LCST, the hydrogen bonds between the polymer chains and water are disrupted resulting in polymer hydrophobicity and precipitation. It is an interesting feature to use the phase change for a controlled destabilization of the polymeric micellar structure and release of payloads (Ward and Georgiou, 2011; Gandhi et al., 2015).

Light-sensitive nanocarriers being non-invasive strategy and with the potential of remote spatiotemporal control have also been widely practiced. Several types of photo-responsive systems have been prepared that release on-demand drug release in response to light of a specific wavelength [in the near-infrared (NIR), visible or ultraviolet (UV) regions]. Photo sensitiveness brought structural changes in the nanocarriers and thus release the encapsulated drugs on demand. For example, the UV–visible reversible photoisomerization of the azobenzene group (and its derivatives) from *trans* to *cis* on irradiation at 300–380 nm, and from *cis* to *trans* by shining light in the visible region enables photo-regulated control of drug release. This phenomenon has been successfully applied for the functionalization of mesoporous silica NPs (MSNPs) pore with azobenzene (Lu et al., 2008) by means of azo-modified DNA valves at the pore mouth (Yuan et al., 2012) and by the light-controlled host–guest recognition between the azobenzene derivatives and cyclodextrin (CD) cavity (Yan et al., 2012).

Magnetic responsiveness release is typically obtained by focusing an extracorporeal magnetic field on the biological target during the injection of a magnetically responsive nanocarrier. This concept has demonstrated great potential in experimental cancer therapy because of improved drug accumulation inside solid-tumor models. Candidate nanosystems for such therapeutic approach are core–shell NPs (a magnetic core made of magnetite (Fe3O_4_) coated with silica or polymer (Hua et al., 2011; Zhang L. et al., 2012), magneto-liposomes (Fe3O_4_) or maghemite (Fe_2_O_3_) nanocrystals encapsulated in liposomes (Plassat et al., 2011) and porous metallic nanocapsules (Zhang F. et al., 2012).

Ultrasound waves can also trigger the release of the drug from a variety of nanocarriers through the thermal and/or mechanical effects generated by cavitation phenomena or radiation forces. It has been shown that physical forces associated with cavitation can induce nanocarrier destabilization, drug release (Schroeder et al., 2009), and transient increase in vessel permeability, leading to the cellular uptake of therapeutic molecules (Kheirolomoom et al., 2010). The use of ultrasound waves is also remarkable because of their non-invasiveness, the absence of ionizing radiations, and the facile regulation of tissue penetration depth by tuning frequency, duty cycles, and time of exposure (Mura et al., 2013).

### Applications of Surface Engineered Nano-Cargos for Antibiotic Resistance

Nanomedicine has emerged into a well-developed medicine designing field by introducing new and innovative alternatives to develop efficient antimicrobial agents to meet the challenge of microbial infections (Colilla and Vallet-Regí, 2020). Most nanocarriers generally encounter at least one of the resistance actions that developed by MDR bacteria. The bactericidal mode of nanocarriers can be predicted from their physicochemical properties, which are distinct for every material (Chen et al., 2014). The nanosize and higher surface area helps NPs to better interact with bacterial cells in contrast to usual antibiotics. Moreover, bacterial cell envelope, which limits the internalization of antibacterial drugs can be effectively overcome by these nanocarriers. The NPs [e.g., metal NPs, carbon nanotubes, dendrimers, cyclodextrin (CD), chitosan (CS), Nnoparticles (Ps), antimicrobial peptides (AMPs)] can directly penetrate the bacterial envelope causing envelope disruption. Some carriers (cell penetrating peptides, siderophores, and CDs) penetrate the bacterial envelope accompanying encapsulated drugs without necessarily causing disruption. Some delivery systems (e.g., fusogenic liposomes) do not penetrate bacteria but intracellularly deliver the drugs into bacteria (Santos et al., 2018).

The anticipation of biofilm formation by interruption of bacterial membranes is another major measure to meet the challenge of bacterial resistance (Peulen and Wilkinson, 2011). The formation of bacterial biofilms serves as a nursery for the interchange of systematic resistance mutations and cause variant of these mutations in various bacterial cells (Khameneh et al., 2016). Serval studies have shown that NPs, such as Au-based NPs (Yu et al., 2016), CuO NPs (Miao et al., 2016), Ag-based NPs (Markowska et al., 2013), Mg-based NPs (Lellouche et al., 2012), and NO NPs (Slomberg et al., 2013), can overcome or prevent biofilm formation; however, the subsequent section summarizes recent advanced functionalized nanosystems applied for overcoming antibacterial resistance.

The key points to be considered in the strategy to design carriers for the precise and sustainable release of antibiotics in antibiotic treatment are: (i) minimizing antibiotic interactions with healthy tissue; (ii) accumulation of antibiotic concentration at the infection site; (iii) enhancing bactericidal treatment efficacy; and (iv) decreasing the toxicity risks and exposure of commensal microflora to antibiotic (Aslam et al., 2018). The approach to develop a stimuli-responsive system that either reacts in a self-motivated way and recognizes the bacterial microenvironment or that is liable to certain physical factors. It is, therefore, one of the probable approaches to develop improved antibiotic delivery systems. It would enhance their efficiency, targeting properties, and the effectiveness of antibiotic treatments while, simultaneously, minimizing the side effects.

Treatment of chronic lung infection emerges as one of the most challenging treatments due to the MDR bacteria. In an attempt to cover this problem, Liu et al. synthesized ciprofloxacin-loaded selenium lipid carriers and investigated for effective delivery of the drug to prevent lung infections (Jin et al., 2019). They adopted a novel approach to develop ciprofloxacin-loaded selenium-lipid NPs (CxLSENPs), and its bactericidal potential was assessed against *Pseudomonas aeruginosa*. The CxLSENPs showed greater antibacterial efficacy against *P. aeruginosa*. For further confirmation of its bactericidal potential and efficiency, they performed live/dead cell assay where the number of dead cells were remarkably higher in CxLSENP-treated groups than the control group. Polymer NPs consisting of poly (DL-lactic-co-glycolic acid) were surface functionalized to deliver nitric oxide (NO). These biodegradable and biocompatible NPs were modified with S-nitrosothiol, S-nitrosocysteamine, as the NO delivery molecules. S-nitrosocysteamine was covalently immobilized on the NP surface. Attachment of the S-nitrosothiol resulted in an NO release of 37.1 ± 1.1 nmol/mg of NPs under physiological conditions and reduced *E. coli* culture growth by 31.8%, indicating that the NO donor was effective at releasing NO even after attachment to the NP surface. Combining the NO-modified NPs with tetracycline for *E. coli* increased the effectiveness of the antibiotic by 87.8%, which allows for lower doses of antibiotics to be used in order to achieve the same effect. The functionalized NPs were biocompatible when tested in mouse fibroblasts cells (Reger et al., 2017). Titania nanotubes (TNTs) were functionalized and designed as specific drug delivery nanosystems for enrofloxacin (Enro). Two kinds of novel drug delivery nanosystems for Enro, that is, Enro-NH2-TNTs and Enro-SH-TNTs, were prepared by combining the characteristic pH-adjusted and surface silane coupling agent-modified TNTs (NH2-TNTs and SH-TNTs). *In vitro* studies of these systems showed excellent controlled-release properties and further proved that the Enro drugs had been loaded into TiO_2_ nanotubes, which were influenced by grafted molecules. These modified drug delivery nanosystems afforded higher drug bioavailability and longer drug effects on *in vivo* administration to chickens (Huang et al., 2014).

In a study, a simple layer-wise spin coating technique has been reported for the preparation of CS/poly-g-glutamic acid (C/PGA) polyelectrolyte multilayers (PEMs) on two different biomedical metals, titanium alloy (Ti6Al4V) and 316L stainless steel (316LSS). The multilayer coating was fabricated using oppositely charged C/PGA to deposit a total of 10, 20, or 30 multilayered films. Then, tetracycline was loaded by soaking the coated metals for 12 h. The microstructure, mechanical properties, biocompatibility, and drug release rate were investigated by SEM, contact angle measurement, MG63 cell viability, and inhibition of *E. coli* growth. The coating technique could prepare a layer of 2.2–6.9 mm C/PGA PEMs favoring cell attachment and growth. Moreover, tetracycline was released from C/PGA PEMs and inhibited the growth of *E. coli*. The results showed a useful platform for modulating the micro-environment for better cell adhesion and antibiotic delivery and could hold a great potential for surface modification and drug loading (Liu et al., 2017).

Inspired by the localized antimicrobial function of neutrophil phagosomes and the versatility of dendrimersomes (DSs), a simple three-component DS-based nanoreactor with broad-spectrum bactericidal activity was prepared. The system was prepared by encapsulating glucose oxidase (GOX) and myeloperoxidase (MPO) within DSs (GOX–MPO–DSs), self-assembled from an amphiphilic Janus dendrimer that possessed a semipermeable membrane. By external addition of glucose to GOX–MPO-DS, the production of hypochlorite (^−^OCl), a highly potent antimicrobial, by the enzymatic cascade was demonstrated. This cascade nanoreactor yielded a potent bactericidal effect against MDR *S. aureus* and *P. aeruginosa*. The production of highly reactive species in response to a bacterial stimulus in a localized manner was also demonstrated using a bacterial trigger (released toxins). The prepared system showed excellent results *in vitro* and could potentially be translated *in vivo* (Potter et al., 2020). Liposomes have successfully improved antibiotic penetration into the Gram-negative *P. aeruginosa, E. coli, Klebsiella* spp., and *Acinetobacter baumannii* (Drulis-Kawa et al., 2006; Nicolosi et al., 2010; Ma et al., 2013) and have intracellularly delivered Nucleid acid mimics (NAMs) into the Gram-negative *Helicobacter pylori* (Santos et al., 2017). Zhao et al. (2010) developed 3 nm AuNPs capped with amino-substituted pyrimidines that exhibited antibacterial activities against MDR clinical isolates. These positively charged NPs effectively bind with and disrupt the bacterial membrane, resulting in leakage of bacterial cell contents, such as nucleic acids (Zhao et al., 2010). Grzybowski et al. (2016) developed mixed-charge NPs with different ratios of positively charged [trimethylamine (TMA)] and negatively charged [11-mercaptoundecanoic acid (MUA)] ligands that exhibit Gram-selective antibacterial activity. NPs with ligand ratio (TMA:MUA) 48:52 and 80:20 had the potential to selectively kill Gram-positive and Gram-negative bacteria, respectively at adequate rates (Grzybowski et al., 2016). In a study, silica NPs surface functionalized with silver/polyrhodanine composite (SiO_2_-Ag/PRh) were found to exhibit long-term antimicrobial activity due to contact-active bactericidal effects of PRh. The combination of positively charged rhodamine on inherently antimicrobial NPs (SiO_2_-Ag^+^) showed a synergistic effect in killing multiple species of bacteria (Song et al., 2013). Shruthi et al. (2019) synthesized a range of silver (Ag) NPs engaging tyrosine, tryptophan, curcumin, or epigallocatechin gallate (EGCG) and functionalized with streptomycin for improved antibacterial activity (). Their results showed that the bacterial viability for both the Gram-positive and Gram-negative strains decreased after treatment with streptomycin-functionalized AgNPs compare to synthesized AgNPs.

Beta-cyclodextrin (β-CD) and its derivatives have been used for the inclusion or association of several antibiotics, such as macrolides, ryfamycins, quinolones, β-lactams, cephalosporins, and tetracyclines, improving the antibiotic potency against Gram-negative bacteria, such as *E. coli, P. aeruginosa, Citrobacter* spp., *Enterobacter* spp., *Klebsiella* spp., and *A. baumannii*, and Gram-positive bacteria, such as *S. aureus* (Athanassiou et al., 2003; Rosa Teixeira et al., 2013; Suárez et al., 2014; Imperiale and Sosnik, 2015; Li et al., 2016). For instance, when tested in *Staphylococcus* spp., *Klebsiella* spp., *E. coli, P. aeruginosa, Enterobacter* spp., and *Citrobacter* spp., β-CD carriers decreased the minimum inhibitory concentration (MIC) of ampicillin and amoxicillin up to four times and the MIC of cefadroxil up to 16 times (Athanassiou et al., 2003). Besides improving the stability and solubility of antibiotics, β-CD and its derivatives were considered to mediate enhanced permeation of the antibiotics. In particular, it has been hypothesized that β-CD may drive internalization of the β-CD–antibiotic complex possibly *via*: (i) enhanced adhesion to the bacterial surface (including pore channels) with potential local release of the antibiotic, (ii) CymA ortholog channels, and (iii) destabilization of the bacterial envelope. In addition, β-CD capping AgNPs improved their interaction at the bacterial envelope of *E. coli, P. aeruginosa*, and *S. aureus* and enhanced the intracellular delivery of antibacterial silver ions (Jaiswal et al., 2010).

Han et al. (2009) constructed nitrite-loaded silane-hydrogel-based composite NP system using tetramethoxysilane (TMOS) as sol–gel matrices required for the thermal conversion of sodium nitrite into NO in the presence of glucose to exploit NO antimicrobial activity. PEG and CS in the composite system served as additives for controlling the generation and sustained release of NO upon exposure to moisture. These NPs demonstrated stable release of NO for up to 24 h and exhibited strong antimicrobial activity against *S. aureus*, particularly, this NO-releasing NP system was able to treat methicillin-resistant *S. aureus* (MRSA) in a murine wound model (Han et al., 2009; Pelgrift and Friedman, 2013). Nanocarriers can also provide a promising co-delivery platform for multiple antimicrobials in single vehicle to get a synergistic effect against MDR bacteria. Wang et al. adopted this tool for co-delivery of Ag and levofloxacin loaded in MSNPs (Wang et al., 2016). They first synthesized Ag-embedded MSNPs and then encapsulated levofloxacin inside the mesopores. The release of Ag^+^ ions made the outer membrane of bacteria more permeable and made bacteria more susceptible to internalization of simultaneously released levofloxacin. This dual antimicrobial-loaded NPs showed synergistic effects against MDR *E. coli* both *in vitro* and *in vivo*. Table 1 summarizes different NP-based antibiotic delivery strategies for skin and subcutaneous infections tested in animals.

**Table 1:** Summary of nanoparticle-based approaches for skin and subcutaneous bacterial infections tested in animal studies.

**Nanoparticle type**	**Drug**	**Bacterial strain**	**Size (nm)**	**Model used**	**References**
Au nanorods	Ag	MRSA	Length: 68; diameter: 21	Mice (Subcutaneous abscess)	Liu et al., 2018
Au	Chitosan	MRSA	8–13	Rabbits (Open wound infection)	Lu et al., 2018
Ag	Ag	*Staphylococcus aureus*	20	Mice (Open wound infection)	Ran et al., 2017
Ag	Allicin and Ag	MRSA	10–30	Mice (Open wound infection)	Sharifi-Rad et al., 2014
SPIONs	Acetylcysteine	*Staphylococcus aureus*	95	Mice (Subcutaneous abscess)	Cai et al., 2019
SPIONs	Clavanin A	*Klebsiella pneumoniae*	10	Mice (Bacteria-containing CVC introduction)	Ribeiro et al., 2018
Liposomes	Chloramphenicol	MRSA	132–239	Nude mice (Skin irritation test)	Hsu et al., 2017
PCL	Carvacrol	MRSA	164–233	Pig skin (Burn wound infection)	Mir et al., 2019
NLCs	SME and oxacillin	MRSA	177	Mice (Subcutaneous abscess)	Alalaiwe et al., 2018
Micelles	SME	MRSA	178	Mice (Subcutaneous abscess)	Yang et al., 2016
MSNPs	Gentamicin	*Staphylococcus aureus*	95	Mice (Subcutaneous abscess)	Yang et al., 2018

*CVC, central venous catheter; MRSA, methicillin-resistant Staphylococcus aureus; MSNPs, mesoporous silica nanoparticles; NLCs, nanostructured lipid carriers; PCL, poly(ε-caprolactone); SME, soyaethyl morpholinium ethosulfate; SPIONs, superparamagnetic iron oxide NPs*.

## Possible Drawbacks of Nanocarriers for Antibiotics

The use of antibiotic-loaded nano-formulations is considered a valid strategy for bacteria targeting because of its numerous advantages over conventional formulations, including improved stability, controlled antibiotic release, targeted capability, and increased bioavailability (Pant et al., 2021). Some issues, such as solubility, drug resistance, and epithelium permeation, can also be resolved by targeting pathogens of nanocarriers. Though many nanocarriers have been developed for testing in cell-based and animal studies, clinical trials for bacteria-delivery applications are still limited. This may be due to the high cost of clinical trials and the unknown side effects that should be first identified and explored (Yeh et al., 2020). Nanosystems are thought to cause more serious adverse effects on organisms compared to the bulk materials, as their very small size causes a correspondingly higher surface area (Navya and Daima, 2016). The researchers should pay attention not only to the therapeutic benefits of NPs but also to their toxic responses to human health. Caution should be taken in optimizing the feasible conditions of nanomedicine for balancing the effectiveness of antimicrobial therapy and tissue damage. For use in future human applications, the materials utilized for preparing antibacterial NPs should be non-toxic, biodegradable, and biocompatible. The materials approved by the FDA may be the first choice for the development of these nanocarriers. There are already some experiences of antibiotic-loaded NPs approved by the FDA for clinical application. To meet this regulatory requirement, some toxic effects of nanomaterials have been evaluated, but according to reports, the toxicological data derived so far are conflicting and inconsistent. Toxicological studies provide a base for the protection of both humans and the environment. Therefore, on the basis of available experimental models, it may be difficult to list some of the more valuable NPs as more toxic to biological systems and vice versa. Considering the potential applications of NPs in many fields and to address the knowledge gap, the relevant toxic effects of NPs should be assessed by utilizing internationally agreed free of bias *in vivo* toxicological models, targeting the vital systems (Bahadar et al., 2016). However, in addition to all, we are of the opinion that designing, adapting, and validating such new models in the future for toxicity testing, route of exposure, coating material and sterility of NPs, and type of cell cultures need to be carefully considered (Navya and Daima, 2016). It is preferable if some suggestions can be made for selecting the NPs with best antibacterial efficiency. However, this intention is difficult to achieve since different investigations involved in the development of antibacterial NPs employ different evaluation platforms. Although the MIC is the assay most frequently used, the protocols of MIC determination are always different among different studies. Thus, it should be cautious to compare the antimicrobial activity of the various types of nanosystem. Nevertheless, the comparison of antibacterial effect of the NPs with a positive control antibiotic approved for clinical application is suggested (Yeh et al., 2020).

## Potential Limitations and Future Perspectives

A range of nano-dimensional materials, including nanorobots and nanosensors that are applicable to diagnose, precisely deliver to targets, sense or activate materials in a live system have been outlined. Initially, the use of nanotechnology was largely based on enhancing the solubility, absorption, bioavailability, and controlled-release of drugs. Even though the discovery of nanodrugs deals with high levels of uncertainties, the discovery of pharmacologically active compounds from natural sources is not a favored option today, as compared to some 50 years ago; hence, enhancing the efficacy of known natural bioactive compounds through nanotechnology has become a common feature.

There has been a continued demand for novel natural biomaterials for their quality of being biodegradable, biocompatible, readily available, renewable, and low toxicity. Beyond identifying such polysaccharides and proteins in natural biopolymers, research on making them more stable under industrial processing environment and biological matrix through techniques such as crosslinking is among the most advanced research area nowadays. Polymeric NPs (nanocapsules and nanospheres) synthesized through solvent evaporation, emulsion polymerization, and surfactant-free emulsion polymerization have also been widely introduced. Since the 1990s, the list of FDA-approved nanotechnology-based products and clinical trials has staggeringly increased and include synthetic polymer particles; liposome formulations; micellar NPs; protein NPs; nanocrystals and many others often in combination with drugs or biologics. These are unique carriers that exhibit many functional and structural properties to enable them for antimicrobial drug delivery and therapy. The enhanced antimicrobial potential of nanocarrier-based antibiotics offers an excellent opportunity for the substitution of traditional ones. A detailed knowledge of the cellular uptake mechanism is of utmost significance for the development of more potent nano-antibiotics. So far, no FDA-approved nano-antibiotic has been made available for systemic human usage; hence, future research should be directed to elucidate the biological, physicochemical, and pharmaco-toxicological properties of nano-antibiotics to develop safe and effective products. Though it looks like a challenging task, the combination of nanocarriers loaded with antibiotics and subsequent surface functionalization for targeting against drug-resistant bacterial species will be an efficient strategy in near future to combat MDR bacteria and prove a game changer in the field of nanomedicine. In short, the arena of nano-antibiotics will serve as the next-generation therapeutics for mitigation of the threat imposed by superbugs. Quality of life will be improved through committed efforts and cutting-edge research in the field of nano-antibiotics.

## Conclusion

The rise and emergent of MDR bacteria have turn out to be a deadly threat to health and become a tough challenge worldwide to be addressed. Understanding the actions of resistance would assist us to monitor and treat the infections caused by MDR bacteria. Biofilm formation, overexpression of efflux pump, genetics alteration at a molecular level, and development of resistance by transferable genetic elements are the crucial resistance actions of bacteria against antibiotics. Several approaches have been adapted to get rid of microbial resistance, including the use of natural bactericidal agents, emerging new antibiotics, employing combination therapy, and also developing NP-based antibiotics nanocarriers. The latter draws more consideration; intensive research has been carried out on the suppression of microbial resistance by developing nanoparticulate systems. Metal-based NPs, polymeric NPs, lipid-based NPs, and NO-releasing NPs are the main nanoparticulate systems that have been designed and employed in this field. It can be concluded that the microbial resistance may be addressed by using the above-mentioned approaches, like the incorporation of natural bactericidal agents into NPs or encapsulating antibiotics alone. However, toxicological evaluations of engineered nanostructures are rather unclear or poorly understood limiting their full potential as nanomedicine. Therefore, toxicological concerns of functionalized nanomaterials need to be fully elucidated in well-established *in vitro* and *in vivo* models for short-term and long-term effects. Moreover, a thorough and detailed understanding of interactions of nanocarriers with biological systems is required to construct nanomaterials with favorable physicochemical characteristics, which will enable them more responsive to different biological environments for therapeutic benefits without any deleterious impact.

## Author Contributions

XY designed the project. WY did literature survey and wrote initial draft. YQ reviewed and corrected the draft manuscript. YY and ZX supervised the project and finalized the manuscript. All authors contributed to the article and approved the submitted version.

## Conflict of Interest

The authors declare that the research was conducted in the absence of any commercial or financial relationships that could be construed as a potential conflict of interest.
